# Can Urea 10% Promote Photosensitizer Uptake Before MAL‐PDT for the Treatment of Facial Actinic Keratoses? Results of a Randomized Clinical Trial

**DOI:** 10.1111/phpp.70058

**Published:** 2025-11-04

**Authors:** Grazia Linda Artelli, Isacco Cattaneo, Luca Rubelli, Cesare Ariasi, Stefano Bighetti, Cesare Tomasi, Gaetano Licata, Simone Soglia, Piergiacomo Calzavara‐Pinton, Mariachiara Arisi

**Affiliations:** ^1^ Department of Dermatology University of Brescia Brescia Italy; ^2^ Department of Experimental and Applied Medicine University of Brescia, ASST Spedali Civili di Brescia Brescia Italy; ^3^ Department of Dermatology Sant'antonio Abate Hospital Trapani Italy

**Keywords:** actinic keratoses (AKs), keratolytic pretreatment, MAL‐PDT, methyl aminolevulinate (MAL), photodynamic therapy (PDT)

## Abstract

**Background:**

Actinic keratoses (AKs) are precancerous lesions that may progress to squamous cell carcinoma (SCC). Photodynamic therapy (PDT) with methyl aminolevulinate (MAL) is an established treatment, often preceded by mechanical curettage to enhance photosensitizer penetration. However, curettage is associated with pain and discomfort, necessitating alternative pretreatment strategies, also applicable in daylight PDT.

**Methods:**

Thirty‐six patients with symmetrical facial AKs were randomized to receive MAL‐PDT on two contralateral areas: one pretreated with a 10% urea‐based keratolytic compound (UBC) for 14 days and the other untreated (control). Protoporphyrin IX (PpIX) fluorescence, clinical outcomes, cosmetic results, and patient satisfaction were assessed. Statistical analyses included the Wilcoxon, Mann–Whitney, and chi‐squared tests (*p* ≤ 0.05).

**Results:**

The urea‐pretreated group showed significantly higher fluorescence intensity (median: 7 [5–9]) vs. controls (median: 5 [3–6]; **p < 0.0001**), indicating improved MAL penetration. Both groups had significant AK reductions (**p = 0.02**). The reduction in Olsen grade I AKs was greater with UBC (**p < 0.0001**), while no significant differences were observed for grade II lesions. Tolerability and patient satisfaction were high, with no significant differences in pain scores, local skin reactions, or cosmetic outcomes.

**Conclusions:**

Pretreatment with a 10% UBC enhances PpIX fluorescence and improves efficacy in grade I AKs when compared to no pretreatment. Thus, it provides a non‐invasive pretreatment option with good efficacy in thin AKs, along with good patient satisfaction and safety.

## 1 Introduction

Actinic keratoses (AKs) are pre‐malignant skin lesions induced by the accumulation of UV‐genotoxic DNA in keratinocytes. As every AK may progress to squamous cell carcinoma (SCC), there is a general agreement [1] that their removal and prolonged follow‐up are encouraged, despite their clinical and histological grade [2].

Several treatment options are available [1]: in case of multiple lesions, it is likely that keratinocytes of the surrounding sun‐damaged skin, the so‐called “field of cancerization”, harbor genotoxic damages. Therefore, treatment approaches aiming to treat the whole area are suggested.

Methyl aminolevulinate photodynamic therapy (MAL‐PDT) is widely utilized as a targeted therapeutic approach for managing AKs and the skin field of cancrization [3] which involves the activation of a photosensitizer by visible light to produce reactive oxygen species within target cells, resulting in their destruction [4]. Initial steps to clean and prepare the lesions are followed by the application of MAL, a precursor of the endogenous photosensitizer protoporphyrin IX (PpIX) that is involved in the biosynthetic pathway of heme. PpIX, in the presence of oxygen and appropriate wavelengths of light, is activated and generates free radicals and reactive oxygen species that are toxic to cells (leading to cellular apoptosis, autophagy, or necrosis).

In traditional PDT, the manual removal of scales and crust (called debridement, or curettage) is fundamental because it facilitates the penetration of the photosensitizing agent (MAL). This procedure involves the physical scraping or removal of hyperkeratotic tissues and lesions with a curette or curette ring, and is particularly recommended for the treatment of superficial basal cell carcinoma (sBCC) and AKs, as it improves treatment outcomes [5, 6].

Despite its advantages in tumor delineation and cost‐effectiveness, curettage is not always well tolerated by patients as it can lead to pain, bleeding, and post‐procedural discomfort. The impact of curettage on healing remains uncertain, particularly on the face and scalp. Last, manual curettage is less convenient in the context of daylight PDT, where a topical method for lesion preparation could facilitate home‐based daylight PDT treatments more easily. One possibility for a topical preparatory method might be to use a keratolytic agent such as urea to increase permeability of the stratum corneum. The present study aims to evaluate the efficacy, safety, and patient tolerability of PDT performed on hemifields of cancerization on the face, after daily application of a 10% urea‐based compound (UBC), and in the absence of any curettage.

## 2 Materials and Methods

### 2.1 Inclusion and Exclusion Criteria

Adult patients (skin phototypes I‐IV) with multiple noninflamed, non‐pigmented I to II AKs of the face were enrolled in the study. All of the AKs had a rather symmetrical distribution on the face, and diagnosis was assessed visually and with the use of dermoscopy. Exclusion criteria were: concomitant clinically significant unstable medical conditions, active severe systemic infectious diseases, drug abuse or alcoholism, current participation in another clinical study, known allergies to any molecule in the study drugs, use of photosensitizing drugs, pregnancy or lactation, any other dermatological disease in the treatment area or a distance of 3 cm, prior topical or physical treatment for AKs within 6 months, and likelihood of poor compliance.

The study was performed at the Dermatologic Department of the University of Brescia from November 2023 to August 2024 by the Declaration of Helsinki, and was approved by the Local Ethic Committee (protocol number 3718). All patients were given verbal and written information on the nature of the study, and signed the informed consent form before enrollment.

### 2.2 Treatment Procedures

At baseline (V0), researchers collected demographic and medical data, including date of birth, sex, weight, ethnicity, Fitzpatrick skin type, general health status, and history of skin diseases, with a particular focus on AK and any prior treatments undertaken in recent years. Two contralateral and symmetrical target areas on the face harboring at least 5 AKs were selected. Randomization with a 1:1 allocation ratio to the treatment options was randomized in a 1:1 ratio, using a computer‐generated list using random permuted blocks of six. Patients and treating physicians were not blinded to group assignment.

The number of AKs was counted for each side of the face, and AK thickness per Olsen grading [5] was recorded. Patients were instructed to apply ~0.5 mg per cm^2^ of emollient cosmetic cream containing 10% urea, shea butter, glycerin, mannose, and thermal exclusively on the selected half of the face at night for 14 days, while the other half was left untreated as a control. After 14 days, MAL‐PDT was delivered in a single session (V1), according to the protocol status of the European Medicines Agency [4]. No mechanical curettage was performed in any area. MAL (16% ALA cream) was applied. After the 3‐h MAL incubation period, the intensity of PpIX fluorescence was measured using a Wood lamp with a peak wavelength of 365 nm (Sunlamp 70 Wood, JELOSIL SRL, Milan, Italy). Fluorescence intensity was documented in Arbitrary Units on a scale from 1 to 10, based on standardized photographic imaging assessed by a blinded evaluator, ensuring an objective measurement of PpIX activation.

Treatment illumination was delivered using a monochromatic red light (635 nm, Atilite CL128 lamp, Galderma Inc), as validated for topical PDT [7]. Pain experienced during PDT irradiation was evaluated using a visual analog scale (VAS) ranging from 0 (*no pain*) to 10 (*worst imaginable pain*).

At a follow‐up visit 3 days after PDT (V2), local skin reactions (LSR) were carefully assessed by documenting the following signs: erythema, desquamation, crusting, edema, vesicles/pustules, and erosions/ulcerations on a graduated scale from 0 (*none*) to 4 (*severe*), with a maximum cumulative score of 24 [6].

**FIGURE 1:**
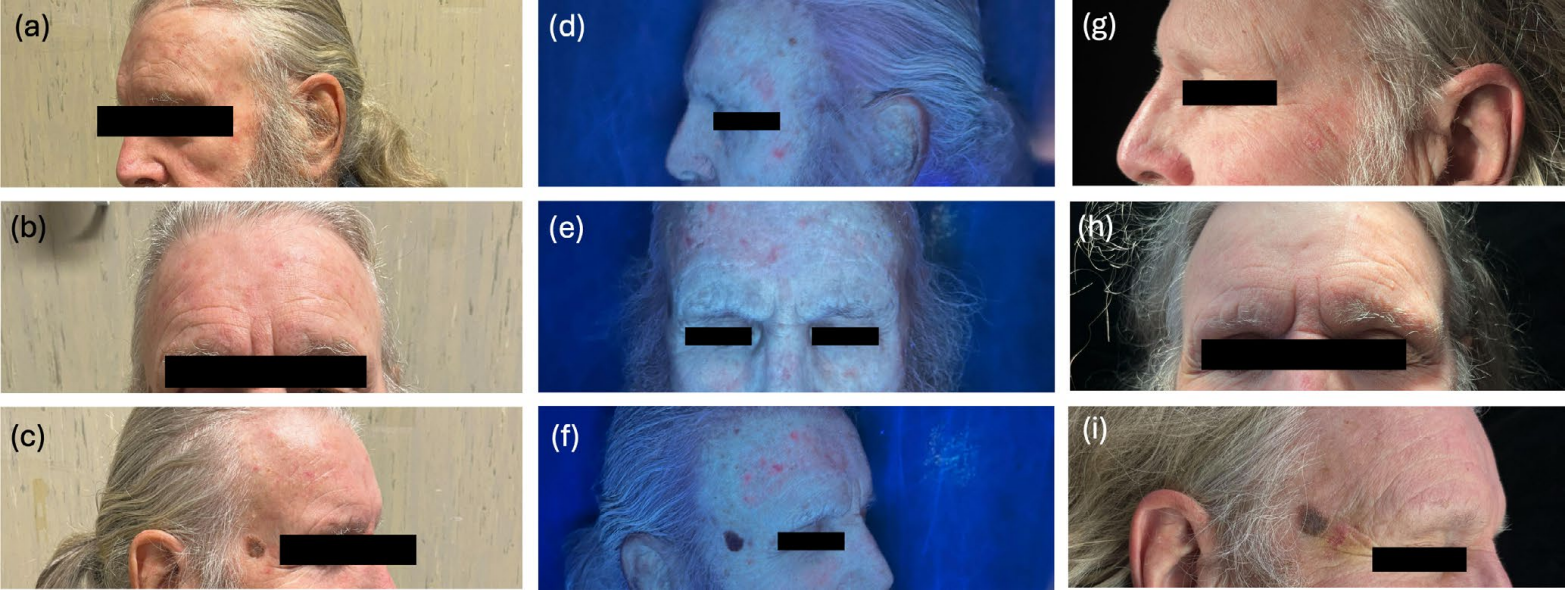
Clinical evaluation in a patient at baseline (V0, a–c. Urea emollient applied on the right side). Fluorescence intensity with Wood Lamp for AK at V1 (d–f). Evaluation of AK lesions post‐treatment (V3; g–i).

One month after PDT (V3), residual AK lesions were counted on each side of the face to assess the treatment's efficacy in reducing lesion numbers. Cosmetic outcome was assessed by an investigator (A.C.) blinded at the initial treatment allocation, and graded into one of four categories: excellent (no or mild redness or change in skin pigmentation), good (moderate redness or change in skin pigmentation), fair (slight to moderate scarring, atrophy, or induration), or poor (extensive scarring, atrophy, or induration) [6]. Patients completed an anonymous questionnaire to report their overall preference using a 5‐point Likert scale (*5PLS*), ranging from very dissatisfied (score 1) to very satisfied (score 5), thus providing insight into how patients perceived the intervention's benefits (Figure 1).

### 2.3 Statistical Analysis

Statistical analysis was performed starting from the formatting of the database in Excel for import use versus IBM‐SPSS software ver. 26.1. Normal distribution of collected data was analyzed by the Kolmogorov–Smirnov test. Categorical variables were summarized by using percentages and continuous variables by calculating medians and ranges (minimum and maximum values). Medians and continuous variables were compared by using the Wilcoxon test and Mann–Whitney test. The chi‐squared test was used for percentage comparison. All results were considered statistically significant at the *p* ≤ 0.05 level.

Based on preliminary data [8, 9, 10] a hypothesis was formulated that 14‐day premedication with 10% urea would result in a 40% difference in fluorescence area (evaluated with Wood's lamp) between the premedicated and non‐premedicated areas. A total of 48 half‐faces (from 24 subjects) were required to achieve a statistical power of 83%.

## 3 Results

### 3.1 Patients

Thirty‐six patients were enrolled in the study. The median (range) age was 80 (57–91) years. Males were 29 (80.6%) and females were 7 (19.6%). Three (8.3%), 28 (77.8%), and 5 (13.9%) had skin phototypes I, II, and III, respectively. All patients completed the study protocol. Kolmogorov–Smirnov test showed that the population was not normally distributed (*p* < 0.05).

The study involved 833 treated lesions, evenly distributed between PDT with 10% urea and PDT alone, with a similar baseline distribution of AKs according to Olsen's grade in both groups. However, fluorescence intensity measured with Wood's lamp was significantly higher in the group treated with 10% urea, suggesting enhanced penetration of the photosensitizing agent (*p* < 0.0001) (Table 1).

**TABLE 1:** Number of lesions treated and fluorescence intensity of both groups.

	10% Urea + PDT	PDT	*p*
Total number of AK	419	414	0.93
Olsen I AK	307	291	0.5
Olsen II AK	112	123	0.48
Fluorescence intensity	7 (range 5–9)	5 (range 3–6)	< 0.0001

**FIGURE 2:**
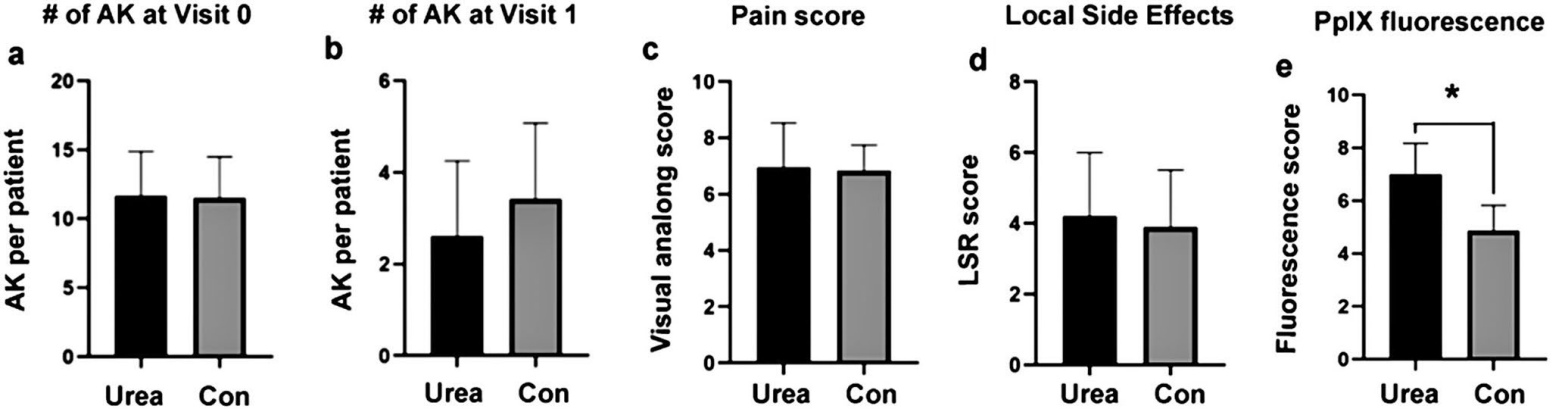
Comparison between the urea‐pretreated areas and the control areas. (a) Number of AKs in the two areas (Urea‐pretreated area and Control) at time V0. (b) Number of AKs in the two areas (Urea‐pretreated area and Control) at time V1. (c) Pain score in the two areas (Urea and Control). (d) Comparison of side effects between the two areas. (e) PpIX fluorescence between the two areas.

**FIGURE 3:**
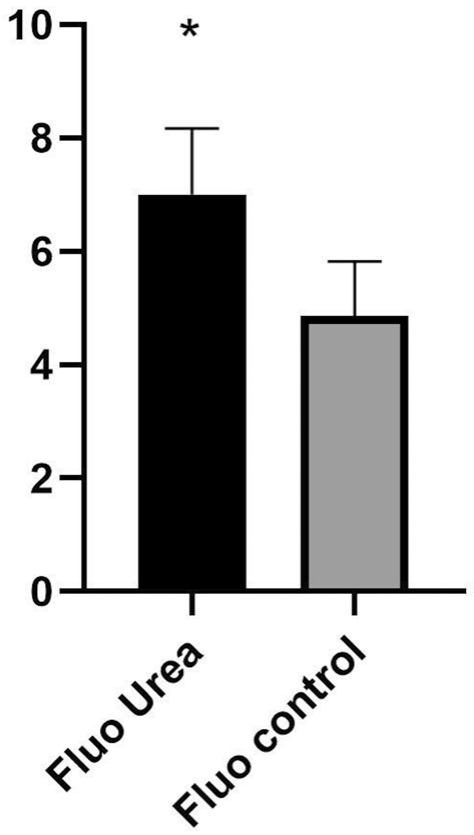
Comparison between fluorescence in the urea group and controls.

Total AKs number and area reduced significantly after 10% urea + PDT and PDT alone (*p* = 0.02). To compare the efficacy of the two treatment modalities, the variation (ΔV0–V3) of AKs number was compared. No significant differences were found (*p* = 0.13) (Figures 2 and 3).

However, the variation (ΔV0‐V3) of Olsen I AKs was more consistent (85%) in the skin areas pre‐treated with urea (total Olsen I AKs at T3 47 vs. 81; *p* < 0.0001). No differences were found for Olsen II AKs' variation, whose percentage has nonetheless decreased by 58% (total Olsen II AKs at T3 47 vs. 42; *p* = 0.24).

The acceptability and tolerability of PDT in terms of pain perceived by the patients (assessed with pain VAS score, Visual Analog Scale 0–10) were deemed excellent or good by all patients who completed the study (Figure 2c). The median pain score was 7 (range 5–9) in both the 10% urea treatment group and the control group; the difference in VAS scores between the two sides was not statistically significant (*p* = 0.44). The local skin reaction (mean LSR score) was 4 (range 2–10) in the 10% urea + PDT areas and 3.5 in the control group (range 2–9), with the highest scores in erythema (Figure 2d). The difference in LSR scores between the two groups was not statistically significant (*p* = 0.41). Patients did not report any other local or systemic adverse events after the two procedures.

The cosmetic outcome was not different at a statistical level of significance (*p* > 0.05); data are disponible to be consulted on Supporting Information.

## 4 Conclusions

Curettage prior to PDT is a well‐accepted pretreatment known to improve treatment outcomes, but it can have significant drawbacks, including bleeding, oozing, and pain [11]. Given these potential problems with curettage, there is value in investigating other methods aimed at reducing the hyperkeratosis of lesions and ensuring maximal effectiveness of PDT while minimizing side effects [12].

The present study aimed to evaluate and compare the efficacy (based on clinical assessments), safety, and patient satisfaction of traditional PDT in the treatment of AKs on the face and related field cancerization using a split‐face design. In this protocol, each patient served as their own control, with one side of the face treated with keratolytics (10% urea) and the other side left untreated before PDT application.

Urea pretreatment resulted in increased fluorescence intensity of protoporphyrin IX (PpIX), indicating improved MAL penetration and activation [13]. Clinically, the median reduction in AK lesions was greater in the urea‐treated group compared to the control group, particularly for grade I AKs, underlining the efficacy of keratolytic agents in facilitating MAL absorption [14]. Importantly, aesthetic satisfaction, treatment tolerability, and patient compliance were high in both groups, with no statistically significant differences observed in these outcomes. These results suggest that a gentle keratolytic pretreatment may represent a viable and effective alternative to curettage to improve the effectiveness of PDT in facial AK, offering a less invasive approach for the patient without compromising the effectiveness of the treatment [15].

These results suggest that a gentle keratolytic pretreatment may serve as a potential alternative to curettage to improve the effectiveness of PDT in facial AK. However, since urea 10% was not directly compared to curettage in this study, further research is needed to evaluate its efficacy relative to this standard approach [16]. Furthermore, some studies have demonstrated that patients prefer home‐based treatments for preparing the skin to treat AKs [17]. Future studies should compare urea compounds directly to curettage to assess its efficacy as a pretreatment method for PDT. Additionally, investigating its potential role in home‐based daylight PDT could offer a more convenient and less invasive alternative for patients.

## 5 Study Potentials and Limitations

Based on the collected data, this study demonstrated that pretreatment with 10% urea is more effective than no pretreatment in enhancing PDT efficacy for facial AK. However, a major limitation is the lack of direct comparison with curettage, which remains the standard physical pretreatment. Therefore, while the results indicate that keratolytic pretreatment can facilitate MAL absorption and lesion reduction, it is not currently possible to confirm that urea‐based conditioning (UBC) can fully replace curettage in terms of efficacy. Future research should include direct comparative trials between UBC and curettage to establish its potential as a less invasive but equally effective alternative.

## Conflicts of Interest

The authors declare no conflicts of interest.

## Supporting information


**Data S1:** Flowchart.

**Data S2:** phpp70058‐sup‐0002‐Supinfo2.docx.

## Data Availability

The data that support the findings of this study are available from the corresponding author upon reasonable request.
