# Supplementary figures and images for: Can Urea 10% Promote Photosensitizer Uptake Before MAL‐PDT for the Treatment of Facial Actinic Keratoses? Results of a Randomized Clinical Trial
Source: Photodermatol Photoimmunol Photomed. 2025 Nov 4;41(6):e70058. doi: 10.1111/phpp.70058 (PMC12585119; doi:10.1111/phpp.70058)

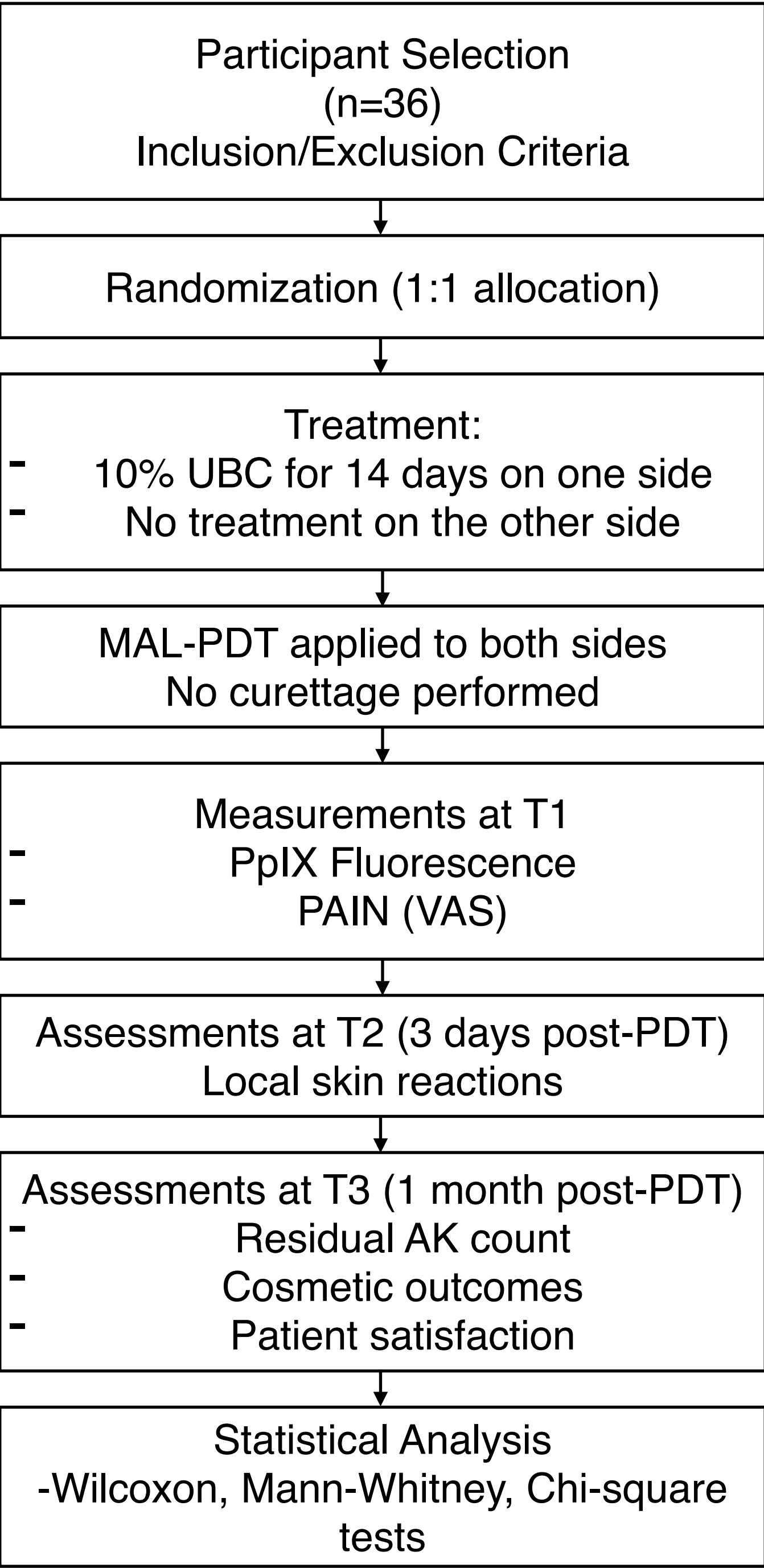

Supplement: Supplementary file 1 — Data S1: Flowchart. [file PHPP-41-e70058-s001.pdf]
