# Supplementary material for: Can Urea 10% Promote Photosensitizer Uptake Before MAL‐PDT for the Treatment of Facial Actinic Keratoses? Results of a Randomized Clinical Trial
Source: Photodermatol Photoimmunol Photomed. 2025 Nov 4;41(6):e70058. doi: 10.1111/phpp.70058 (PMC12585119; doi:10.1111/phpp.70058)
Supplement: Supplementary file 2 — Data S2: phpp70058‐sup‐0002‐Supinfo2.docx. [file PHPP-41-e70058-s002.docx]

**SUPPLEMENTARY TABLE 1a and 1b**

**1a. Cosmetic outcome**

| **Treatment Group** | Ratings | Number of Patients | Percentage |
| --- | --- | --- | --- |
| **10% Urea + PDT** | Excellent | 18 | 50% |
| **PDT only** | Excellent | 19 | 52.8 |
| **10% Urea + PDT** | Good | 18 | 50% |
| **PDT only** | Good | 17 | 47.2% |

**1b. Patients’ satisfaction**

| **Treatment group** | Total 5PLS score |
| --- | --- |
| **10% Urea + PDT** | 382 |
| **PDT only** | 395 |

**SUPPLEMETARY FIGURES**


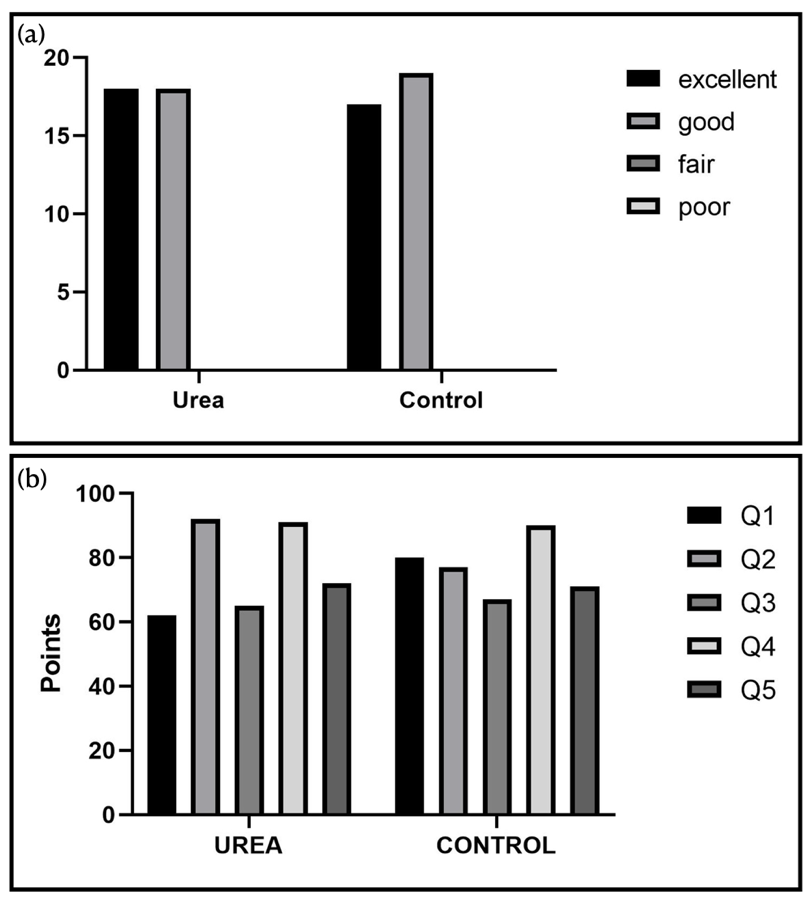


**1a.** Cosmetic outcomes following treatment with 10% urea + PDT (UREA) and PDT alone (CONTROL). Outcomes were rated by patients as Excellent or Good. In the UREA group, 18 patients rated the result as Excellent and 18 as Good. In the CONTROL group, 17 rated it as Excellent and 19 as Good. No patients in either group reported Fair or Poor outcomes.

**1b.** Patient satisfaction scores based on the 5-point Likert scale (1 = Strongly agree to 5 = Strongly disagree), with lower scores indicating higher satisfaction. The graph compares responses from patients treated with 10% urea + PDT (UREA) and PDT alone (CONTROL) across five domains: ease of use (Q1), healing time (Q2), cosmetic outcome (Q3), satisfaction compared to previous treatments (Q4), and overall satisfaction (Q5). Patients in the UREA group reported higher satisfaction for Q2, Q4, and Q5, while the CONTROL group scored higher in Q1 and Q3.
